# Development of Active Centrifugal Pump for Microfluidic CD Platforms

**DOI:** 10.3390/mi11020140

**Published:** 2020-01-27

**Authors:** Ala’aldeen Al-Halhouli, Baha El Far, Ahmed Albagdady, Wisam Al-Faqheri

**Affiliations:** 1 NanoLab, School of Applied Technical Sciences, German Jordanian University (GJU), Amman 11180, Jordan; ahmed.albagdady@gju.edu.jo (A.A.); walfaqheri@chipcare.ca (W.A.-F.); 2 Institut für Mikrotechnik, Technische Universität Braunschweig, 38106 Braunschweig, Germany; 3 Faculty of Engineering, Middle East University, Amman 11831, Jordan; 4 School of Engineering and Technology, Central Michigan University, Mount Pleasant, MI 48859, USA; Elfar1b@cmich.edu

**Keywords:** microfluidics, lab-on-disc, micro pumping, centrifugal pumps

## Abstract

The continuous emerging of microfluidic compact disc (CD) platforms for various real-life applications motivates researchers to explore new innovative ideas towards more integrated active functions. However, microfluidic CDs have some drawbacks, including the unidirectional flow that limits the usable space for multi-stepped biological and chemical assays. In this work, a novel active and bidirectional centrifugal pump is developed and integrated on microfluidic CDs. The design of the developed pump partially replicates the designs of the conventional centrifugal pumps with a modification in the connecting channels’ positions that allow the developed pump to be reversible. The main advantage of the proposed centrifugal pump is that the pumping speed can be accurately controlled during spinning or while the microfluidic CD is stationary. Performance tests show that the pumping speed can reach up to 164.93 mm^3^/s at a pump rotational speed (impellers speed) of 4288 rpm. At that speed, 1 mL of water could be pumped in 6.06 s. To present a few of the potential applications of the centrifugal pump, flow reciprocation, bidirectional pumping, and flow switching were performed and evaluated. Results show that the developed centrifugal pump can pump 1096 µL of liquid towards the CD center at 87% pumping efficiency while spinning the microfluidic CD at 250 rpm. This novel centrifugal pump can significantly widen the range of the applicability of microfluidic CDs in advanced chemical processes and biological assays.

## 1. Introduction

For the last few decades, the ability to miniaturize complex processes using lab-on-a-chip (LOC) technology has drawn great attention from researchers in the chemical, pharmaceutical, and biological fields [1,2,3,4]. Compared to traditional analysis tools, LOC can achieve highly sensitive and reliable results with improved processing time, reduced sample volume, higher flexibility, and portability [5]. With such integration, flexibility, and automation, LOC can perform multi-stepped assays without the need for a skilled operator, minimal or no laboratory tools, and simple operational protocols that make results more reliable with minimum human error. In such highly miniaturized and integrated platforms, the accuracy of liquid manipulation (mainly pumping and valving) can form the main factor in the repeatability and accuracy of the final achieved results [6,7]. Therefore, a large number of liquid manipulation strategies have been developed and tested that can be categorized as passive and active methods. Passive manipulation methods mainly rely on microfluidic design, microchannels geometry, and internal forces to effect liquid flow without the need for external forces or triggers. On the other hand, active methods utilize an external force field for liquid manipulation. Numerical and experimental studies have been devoted to study and understand the hydrodynamical behavior of the fluid in similar devices using passive or active methods [8,9,10,11].

Microfluidic compact discs (CDs) are disc-shaped platforms with networks of microchannels and chambers [12]. In contrast to other microfluidic platforms, microfluidic CDs exploit the centrifugal force to pump the liquid, and capillary force to control the flow. The flow rate and direction can be controlled by controlling the spinning speed of the microfluidic CD. By implementing this pumping method, only a compact and accurately-controlled motor is required, without the need for external syringe pumps [13]. This can also eliminate environmental contamination and minimize air bubbles that are usually introduced with the utilization of external pumping mechanisms. However, the permanent radial outward direction of the centrifugal force can limit the range and complexity of applicable processes [14]. As a result, various passive and active pumping methods were developed to pump the liquid towards the spinning center (microfluidic CD center).

Most passive pumping methods have relied on the pneumatic force generated from compressing trapped air at high spinning speeds [15,16,17]. The compressed air can propel the liquid back towards the microfluidic CD center when the spinning speed is rapidly dropped to low or zero rpm. The limited volume of liquid that can be pumped by passive pumps at one cycle can be considered the main disadvantage of these methods. Therefore, active pumping methods were proposed to tackle some of the passive pumping limitations. For example, an external heating source (hot air, or UV light) was proposed to generate expansion for a trapped volume of air to pump a preloaded liquid towards the CD center [18,19,20,21]. However, this method can damage the loaded biological samples or reagents, and can cause deformation of the microfluidic CD materials when exceeding a specific threshold. To solve this problem, an externally focused airflow to pump the liquid inwards has been proposed [22]. However, this can be a massive source of contamination when used in a contaminated or outdoor environment.

Haeberle et al. [23] enclosed an active pump that utilizes an external magnetic field to manipulate two integrated steel plates integrated on the microfluidic CD. Steel plates are mounted in a PDMS flexible layer and aligned on top of two chambers: a pump chamber, and a valve chamber. As microfluidic CD is spun at a specific frequency on top of the externally fixed permanent magnets, steel plates start to fluctuate in a specific frequency to achieve active pumping or valving mechanisms to continuously and efficiently pump liquid and air. In spite of the different advantages of this proposed method, increasing the pumping rate requires increasing the spinning speed of the microfluidic CD. This spinning increment can prematurely actuate other microfluidic processes, such as passive valves. In addition, the dragging force between permanent magnets and the mounted steel plates can interrupt the spinning speed stability of the microfluidic CD. 

As a result, active, efficient, enclosed, and continuous pumping methods need to be developed to conduct multi-stepped applications on the microfluidic CD. Hence, in this paper, a novel microfluidic centrifugal pump has been designed, fabricated, and tested on a microfluidic CD to overcome all the previous challenges, along with adding major contributions in fundamental applications of microfluidic CDs. This active pumping method is efficient (could reach 87%), easy to fabricate, inexpensive, practical for large-scale and it also can easily control the flow rate and flow direction. In continuous flow pumping, the pump could pump 1 mL of water in 6.06 seconds when rotating at 4288 rpm. Additionally, this microscale pump is not limited to the speed of the microfluidic CD as the method of pumping is separated from the microfluidic platform’s speed; therefore, it can pump while the microfluidic CD is stationary, or, while rotating at higher speeds upwards of 400 rpm (without any need to increase or decrease microfluidic CD speed). In this paper, we demonstrate the pump characterizations and performance at various spinning speeds of 200 rpm, 300 rpm, and 400 rpm. Experimental results are also provided. Three fundamental applications for continuous pumping, reverse pumping, and flow switching along with pumping have been tested to verify the pump’s capability and efficiency.

## 2. Design and Operational Concept

### 2.1. Microfluidic Centrifugal Pump

**Figure 1:**
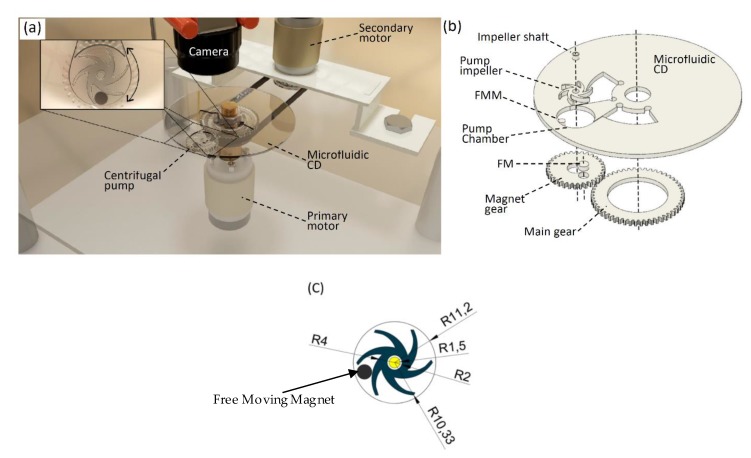
Developed centrifugal pump on microfluidic compact discs (CDs): (**a**) Dual-motor spinning system, mounted microfluidic CD, and integrated centrifugal pump. The top left corner shows a zoomed view of the pump with the free moving magnet installed and the possibility of bidirectional motion for the impeller; (**b**) 3D picture shows the main components of the developed centrifugal pump; and (**c**) the impeller’s design showing the free moving magnet, dimensions in mm.

Centrifugal pumps are employed to transfer the fluid from a source (reservoir) to a desired position by converting the rotational kinetic energy of the fluid to hydrodynamic energy. In most cases, the rotational energy of the pump’s impeller comes from an electric motor or an engine that is connected to the impeller by a drive shaft. In this paper, a small-scale active centrifugal pump on the microfluidic CD is developed to pump the liquid towards the spinning center. However, instead of using an electric motor to drive the pump, magnetic force is utilized. As can be seen in Figure 1a, two main components are implemented: a dual-motor spinning system to spin the microfluidic CD, and a microfluidic CD with an integrated centrifugal pump made from polymethyl methacrylate (PMMA). The dual-motor spinning system was developed and discussed in detail in our previous paper [12]. Briefly, the dual-motor spinning system consists of two DC brush motors (primary and secondary motors) that are controlled simultaneously and accurately by a LabVIEW software (LabVIEW 2014, National Instruments, Austin, TX, USA). A hardware triggered high-speed camera (acA800-510uc, Basler, Ahrensburg, Germany) is used for live monitoring of the process. The primary motor is attached directly to the setup table and is used to spin the microfluidic CD. On the other hand, the secondary motor is inversely positioned on the setup table, using a custom-made holder. This motor is used to spin the main gear that is positioned coaxially under the microfluidic CD. The main gear is connected to the secondary motor using a rubber belt. It transfers the secondary motor rotation to the magnet-gear positioned coaxially below the chamber of the centrifugal pump (magnet-gear and pump impeller have the same spinning center-position) as shown in Figure 1b. The magnet-gear is attached to the microfluidic CD by a fixed shaft joint which is located below the pumping chamber. The impeller’s design is illustrated in Figure 1c. The diameter of the pump plays an important role in determining the flow rate in the channel, however, one of microfluidics’ aims is device miniaturization, and larger pumps might not be realizable on microfluidic discs.

The magnet-gear carries a 6 mm × 3 mm disc permanent magnet fixed into the gear plate (fixed magnet, FM), and is used to magnetically-drag a free moving magnet (FMM) positioned in between the impeller curved vanes (Figure 1a,b). Once the magnet-gear rotates, the position of the FM will change in a circular track, accordingly, this motion will force the FMM to rotate in the same direction (clockwise, CW, or counterclockwise, CCW). The attraction between the two magnets is adequate to rotate the impeller. The main advantage of the developed mechanism that the centrifugal pump can be actuated and controlled during the spinning of the microfluidic CD. In other words, the liquid can be pumped from the outer rim of the microfluidic CD towards the spinning center against the centrifugal force, which results from the spinning of the platform.

In this work, the main gear and the magnet gear are designed and positioned according to the sun and planet gear mechanism. This is to transfer the secondary motor movement to the centrifugal pump impeller (to drive the centrifugal pump). Any planetary gear set [24] consists, mainly, of two meshed gears, the sun gear (main gear) and planet gear (magnet-gear). In addition, the center of the planet gear revolves around the center of the sun gear, through an arm that carries the planet gear; in other words, the arm and sun gears have a fixed axis, while, the planet gear, which is meshed with the sun gear, has a moving axis, rotating by the arm around the center of the sun gear. In this work, the microfluidic CD represents the arm that carries the planet gear. To calculate the angular velocities (rotational speeds and directions) of the sun gear, planet gear, and the arm, the following equation is used [24]:

(1) $$m_{v}=\frac{n_{4,3}}{n_{2,3}}=\frac{n_{4}-n_{3}}{n_{2}-n_{3}}$$

where $m_{v}$ is the speed ratio, $n_{4,3\;\;}$is the relative angular velocity of planet gear to the arm (rev/min), $n_{2,3}$ is the relative angular velocity of the sun gear to the arm (rev/min), $n_{4}$ is the planet gear angular velocity (rev/min), $n_{3}\;$is the arm angular velocity (rev/min), and $n_{2}\;$is the sun gear angular velocity (rev/min). As the angular velocity is a physical vector quantity, both direction and magnitude (speed) are required to define it; if a rotating part (for example, the planet gear) is rotating CCW, the angular velocity should have a positive value, however, if that part is rotating in the CW direction, the value of the angular velocity should be negative. The speed ratio $m_{v}$, on the left side of Equation (1) can be calculated by knowing the gear ratio, $m_{g}$, according to the following equation:

(2) $$m_{v}=\frac{n_{4,3}}{n_{2,3}}=-\frac{1}{m_{g}}=-\frac{N2}{N4}$$

where N4 and N2 are the number of teeth of both planet and sun gears, respectively. The implemented dual-motor spinning system has a spinning speed range of 0–3000 rpm. The primary DC motor is employed for driving the arm (Microfluidic platform), and the secondary motor has been utilized to control the speed of the sun gear. Both the input speeds of the arm and the sun gear can control the speed of the planet gear (pump’s driver), which could be considered the same speed of the microfluidic pump, according to the following equation:

(3) $$n_{4}\;=\left(\;-\;\frac{N2}{N4}\;\times \left(n_{2}-n_{3}\right)\right)+n_{3}$$

**Figure 2:**
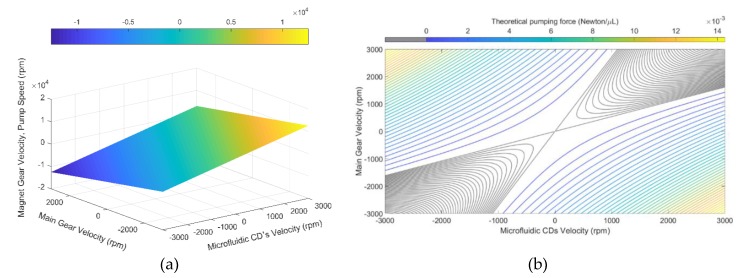
(**a**) Theoretical 3D modeling of the magnet-gear angular velocity (pump speed) with varying both speeds and directions of the main gear and the microfluidic CD; and (**b**) theoretical (ideal) pumping force by the pump as function of the velocities of main gear and microfluidic CD.

By knowing the number of teeth of both main gear (N2) and magnet-gear (N4), which are 54 and 34, respectively, the magnet-gear angular velocity (speed and direction) can be measured and simulated according to the variation of two variables, $n_{3}$ and $n_{2}$, the angular velocities of the microfluidic CD and main gear, respectively. However, those variables are controllable by our dual-motor spinning system, and consequently, controlling both microfluidic CD and main gear actions will lead to controlling the magnet-gear angular velocity (centrifugal pump speed). Figure 2a presents a 3D representation of the magnet gear’s angular velocity while changing both the microfluidic CD and main gear angular velocities from −3000 to 3000 rpm, in which −3000 rpm represents the maximum possible speed of the DC motor in CW direction, and 3000 rpm is the maximum speed of the motors in CCW direction. As shown, the maximum speed (magnitude) that the magnet gear (centrifugal pump speed) can approach is 12,529.4 rpm in both directions. Note that the magnet-gear will reach the maximum possible speed in the CW direction, once the main gear and microfluidic CD rotate at 3000 rpm and −3000 rpm, respectively, and vice versa. The centrifugal force caused by the rotating CD acts against the centrifugal force exerted on the fluid by the rotating impeller, and the summation of these forces returns the theoretical pumping force. Since rotating the microfluidic CD and the main gear in opposite directions results in the highest impeller rotational speed, the highest pumping force is also expected to occur in those regions. Figure 2b demonstrates the theoretical ideal pumping by the pump as a function of the gear and CD rotational speed. Theoretical pumping force per 1 µL of water was plotted using the centrifugal force equation described below:

(4) $$F_{pump}=mV_{im}{}^{2}/r_{im}-mV_{CD}{}^{2}/r_{CD\;}$$

where, $F_{pump}$ is the pumping force, m is the mass (which is assumed to be the mass of 1 µL of DI water), $V_{im}$ is the tangential velocity of the impeller, $V_{CD}$ is the tangential velocity of the CD at the impeller’s tip, $r_{im}$ is the impeller’s radius, and $r_{CD}$ is the distance from the CD’s center to the impeller’s tip. Figure 2b represents the theoretical pumping force for all possible values of rotational speeds of microfluidic CD and main gear, while the grey area in the figure represents the rotational speeds of the CD and main gear that returns a total force that is below or equal to zero. The MATLAB code used to plot the force is provided in the supplementary material.

### 2.2. Microfluidic CD Design and Fabrication

In this work, various microfluidic CD designs were developed and tested. In all tests, the microfluidic CD consisted of five layers; three PMMA layers (Moden Glas, Bangkok, Thailand), and two layers of pressure-sensitive adhesive (PSA) material (FLEXcon, Spencer, MA, USA). The upper 1 mm PMMA contains venting holes, the middle 3 mm PMMA layer has the microfluidic features, including channels, chambers, and pump, while a solid 1 mm PMMA layer was used as the bottom layer to seal the features. Pump impellers and gears were also fabricated using PMMA sheets. The two 200 µm PSA layers are positioned between the PMMA layers for bonding purposes. All PMMA and PSA layers were patterned using a CO_2_ laser cutter (Bodor, Shandong, China). The fabrication process starts with drafting the CAD design of the intended process, then using the CO_2_ laser cutter for micromachining the PMMA and PSA layers. The PMMA layers then go through various cleaning and drying processes to avoid any particle contamination on the CD surfaces to be bonded in a later step. Afterwards, pump impellers, FM and FMM magnets, and gears are put in place, and the CD layers are aligned and bonded together using the transparent pressure-sensitive adhesive (PSA) layers under a custom-made screw press. The pump’s shaft is fixed and sealed in the center of the pumping chamber while the impeller is free to spin with a margin clearance as shown in Figure 1c. There is a hole in the bottom of the centrifugal pump to allow the impeller shaft to pass through it. The shaft is tight in the hole; however, superglue and epoxy were used to seal the shaft to avoid any air leakage. Liquid flow progress was captured using the high-speed camera mounted on the dual-motor spinning system (Figure 1a). The camera can be either synchronized with the primary motor (microfluidic CD) or the secondary motor (centrifugal pump), in which the camera can capture one frame per revolution. This synchronization of the frame rate (frame per revolution) was made to ensure that the desired position (microchannel, chamber, etc.) has been captured at the same location in all captured frames. In addition to this, a custom-made LED lighting system was utilized to optimize the image brightness and clarity. The LED system offers two modes of operation: stroboscope mode (LEDs blink according to the rotations speeds of either microfluidic CD or the pump), and a dimming mode in which the brightness is controlled by a potentiometer. The user interface of the dual-motor spinning system was designed and developed using NI LabVIEW software. This software can provide feedback on the motors’ speeds and directions, a real-time controlling of the motors, as well as an automatic frame rate adjustment. The CAD drawings of the microfluidic features layer from all the CDs fabricated in this work are provided in the supplementary material.

## 3. Results and Discussion

### 3.1. Pump Performance While the Microfluidic CD Is Stationary

**Figure 3:**
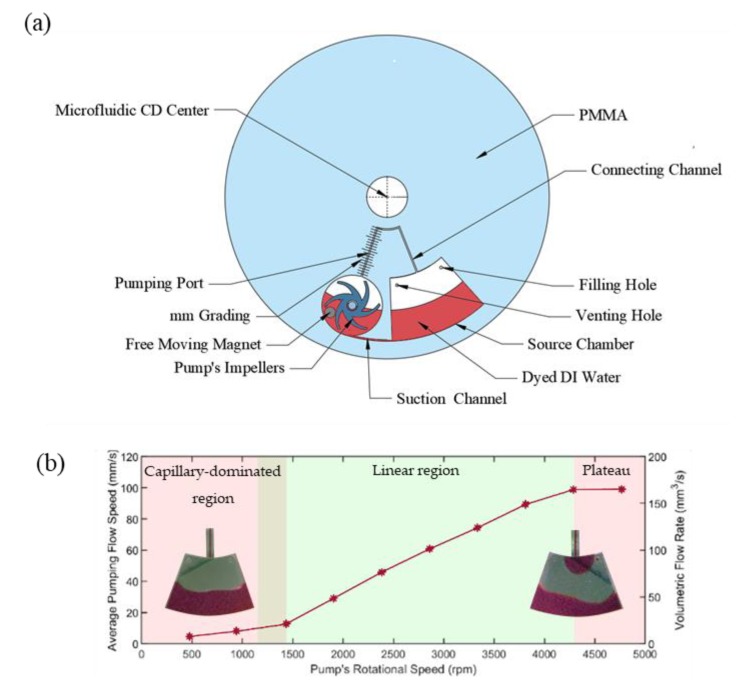
Pumping performance of the developed centrifugal pump. (**a**) Microfluidic design for this test, and (**b**) average pumping flow speed vs. pump speed showing the three flow regions.

In our first experiment, the performance of the proposed centrifugal pump is tested. To perform targeted experiments, a microfluidic design that consists of a source and pumping chambers, centrifugal pump, suction, and pumping channels was designed and fabricated (see Figure 3a). The three components are connected using a 0.5 mm width by 3 mm depth channels. The connection channel between the pump and source chamber is marked with a millimeter-scale to calculate the pumping flow rate using the high-speed camera. The pump performance was observed and measured at a range of pump speeds while the microfluidic CD kept stationary. The pump speed (impeller rpm) is calculated using Equation (3), where the 300–3000 rpm of the secondary motor produces the 476–4764 rpm of the impeller speed. In addition to the performance test, the developed microfluidic design shows one of the microfluidic processes of the developed pump, which is the continuous circulation of liquid from and to the same microfluidic chamber. Figure 3b demonstrates the experimental results of the pumping performance test where the pump flow rate versus the pump speed is recorded and presented. Each experiment was repeated four times (the original data is provided in the supplementary material) and the average standard deviation was 1.2 mm/s. Results confirm the proportional relationship between the pumping flow rate and the magnitude of the pump speed where the flow rate trend increases very gently until the pump speed approach 1430 rpm. Afterwards, the flow rate line increased gradually to reach the maximum pumping speed (109.9 mm/s) at the pump’s rotational speed of 4288 rpm. The sudden expansion from the connecting channel to the source chamber creates a capillary valve, and for the liquid to enter the source chamber, the pressure at the meniscus must be less than or equal to the capillary pressure [25,26]. Since the experiment is performed while the primary CD is at rest, then there are no forces besides the pumping force that drives the fluid further from the CD center, causing the liquid to never enter the source chamber at low impeller speeds as can be seen in the capillary-dominated region in Figure 3b. However, when the pumping force overcomes the capillary force at speeds higher than 1430 rpm, the fluid enters the source chamber directly without stopping. After 4288 rpm, the flow remained constant in spite of increasing the pump’s speed, and it enters the plateau region. The region between 1430 and 4288 rpm is the region where a linear relationship between the flow rate and the impeller speed can be found before it finally plateaus.

### 3.2. Pumping Balance Head Points

The balance head is the maximum point that pumped liquid can be propelled by a centrifugal pump. At this point, the liquid flow rate drops to zero. This is an important indication to find the right pump speed that makes the pumped liquid achieve a targeted height (head). For the presently developed centrifugal pump, the balance head occurs when the pumped liquid faces an opposing force as the centrifugal force, which acts away from the center of the CD. Therefore, finding the right pump speed to each spinning speed of the microfluidic CD is crucial to make sure that the pumping force will always overcome the centrifugal force and liquid will achieve the targeted head. The same microfluidic design in Figure 3a is used to conduct this experiment. The balance head of the proposed pump was tested while varying the spinning speed of the microfluidic CD at three different rpm: 200 rpm, 300 rpm, and 400 rpm. On the other hand, pump speed was increased from zero up to 4364 rpm at each speed of the microfluidic CD and the balance head was recorded until the liquid reached the destination chamber. 

**Figure 4:**
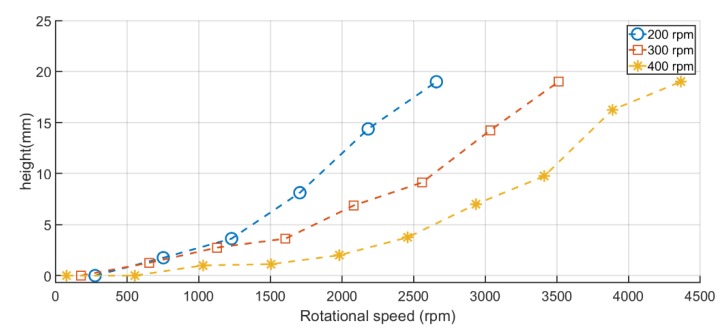
Experimental pump rotation speed vs. pumping balance points while the microfluidic CD is rotating at 200, 300, and 400 rpm.

Figure 4 illustrates the pumping performance while the microfluidic CD is rotating, showing the pumping balance head points in mm with the variation of the pump speed. Each experimental point was repeated four times (n = 4) and results were observed using the high-speed camera. The experimental results show the capability of the centrifugal pump to not only pumping the liquid while the microfluidic CD is stationary, but also while it is spinning at a different range of speeds (100–400 rpm). The pressure can be calculated from Bernoulli’s equation taking one point at the start of the connecting channel and another at the fluid maximum height. The kinetic energy in this case is the same for the two points and it cancels out, therefore, the only difference would be the potential energy, leading to the simplified equation:

(5) $$P=\rho a_{c}h$$

where P is the pressure at beginning of the pumping port, h is the fluid column height, and $a_{c}$ is the centripetal acceleration at the head point, which acts as the gravitation acceleration in conventional systems. 

The results have validated the fact of increasing spinning speed of microfluidic CD will lead in increasing the centrifugal force of the fluid toward CD’s rim. It was observed that when the speed of microfluidic CD increases, higher pumping forces are needed to overcome fluid centrifugal force. The increase in pumping force was done by increasing the pump speed. For this reason, whereas the spinning speed of the microfluidic CD went up, the pumping balancing points were shifted to the right, where higher speeds of the pump are required to achieve the same head at a lower CD speed.

### 3.3. Flow Reciprocation by Continuous Pumping

**Figure 5:**
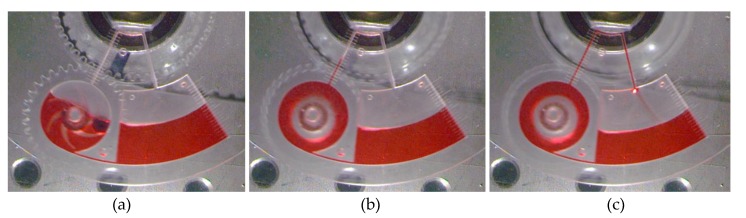
Continous pumping mechanism on a microfluidic CD at 400 spining speed, (**a**) initial state (without pumping), (**b**) during pumping, and (**c**) during fluid’s circulation (Continous pumping).

In this test, the ability of the developed centrifugal pump to generate flow reciprocation will be validated. Flow reciprocation is a very important process in various analytical assays that require multi-wash or mixing steps. When performing flow reciprocation on an antigen-printed microarray, the analyte volume can be significantly reduced from 300 µL to 10 µL, and the processing time can be reduced from 105 min to 10 min [27]. In addition, multi-stepped washing for the printed microarray can be performed when the developed centrifugal pump is integrated with a chamber of washing buffer. Using the microfluidic design in Figure 3a, continuous pumping will be conducted while spinning the microfluidic CD at 400 rpm. However, this process can be implemented while the microfluidic CD is static or spun at higher spinning speed (higher than 400 rpm). The flow circulation rate can be accurately controlled by controlling the spinning speed of pump impellers; once the speed of the microfluidic pump increases, the fluid flow rate in the circulation process increases accordingly. The fluid continuous pumping can work on the whole range of 0–400 rpm spinning speed. Figure 5 presents the pictures of different stages from the real experiments of the performed continuous pumping. The experiment starts with injecting red-dyed water (originally orange-colored but changed to red in the figures for visualization purposes) in the source chamber, the microfluidic CD was spun at 400 rpm. The centrifugation process forces the liquid to level out equally in the pumping and source chambers (Figure 5a). Afterward, the centrifugal pump was initiated, and impellers rpm was increased gradually until the circulation process occurs. At 1030 rpm pumping speed, the fluid started reached a balance point of 1 mm on the microchannel grading scale, while full circulation was observed at 4364 rpm (Figure 5c). The achieved results agree with the experimental curve in Figure 4. It is important to mention here that forces generated by the centrifugation process of the microfluidic CD and centrifugal pump are both important to produce a continuous circulation of liquid. Centrifugal-induced pumping force is important to pump liquid towards the center of the microfluidic CD, while the centrifugal force pushes the liquid back to the source chamber at the prime point. In addition to this, the centrifugal force was another reason to transfer the fluid from the source chamber to the pumping chamber at the initial state. The recorded video for continuous pumping is available in the supplementary material.

### 3.4. Bidirectional Pumping

**Figure 6:**
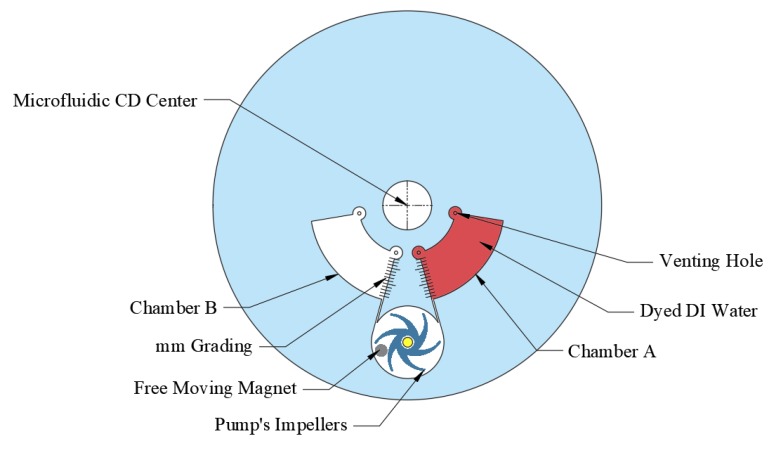
Microfluidic design and experiment for bidirectional pumping.

In this section, the ability of the developed pump to perform bidirectional pumping between two different microfluidic chambers is demonstrated and evaluated. This is an important active pumping mechanism that can be implemented in various multi-stepped applications. To perform this process, a microfluidic design shown in Figure 6 that consists of two chambers (chambers A and B), both connected to the pumping chamber through two connecting microchannels. The two chambers are graded from 0 to 14 mm to visually calculate the pumping performance. Compared to traditional centrifugal pumps where the inlet is usually positioned in the center of the pumping chamber, the connecting channels are positioned as a tangent for the pumping chamber in the developed centrifugal pump. This adjustment enables the bidirectional pumping mechanism by reversing the direction of the impeller rotation (CW or CCW). 

**Figure 7:**
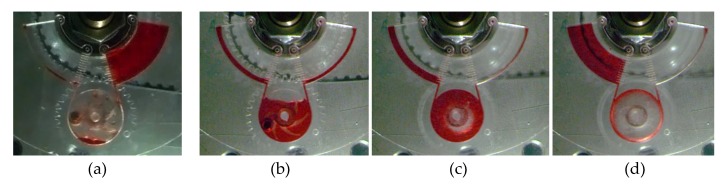
Microfluidic CD (**a**–**d**) bidirectional pumping progress, showing the liquid transferring from chamber A to B. (**a**) the initial state, (**b**) liquid transfer to pumping chamber by rotating the microfluidic CD, (**c**) activating the pump to transfer the liquid to chamber B, (**d**) complete liquid transfer to chamber B.

The experiment starts with loading chamber A with 1096 µL of red-dyed water which makes the top level of liquid reach the 14 mm grading. Afterward, microfluidic CD is spun and kept at 250 rpm to prime the microfluidic channel (reach burst frequency) where liquid transfers from chamber A to the pumping chamber. To pump the liquid towards chamber B, the centrifugal pump is activated at a range of speeds while the microfluidic CD continues spinning. The process can be reversed by simply stop the centrifugal pump to let the liquid flow back towards the pumping chamber and then activating the pump in the reverse direction to pump the liquid back to chamber A. Figure 7a–d presents pictures for the four main stages to pump the liquid from chamber A to B during the real experiment.

It can be noticed that when liquid bursts from chamber A to the pumping chamber, around 200 µL stays in chamber A and is generated in chamber B due to the large amount of loaded liquid that cannot be fully accumulated in the pumping chamber. This passively generated amount of liquid is always theoretically subtracted when calculating pump performance. To calculate the pumping performance in both directions, the following equation is utilized [16]:

(6) $$\eta_{pumping}=\frac{pumped\;Volume}{Total\;Initial\;Sample\;Volume}$$

**Figure 8:**
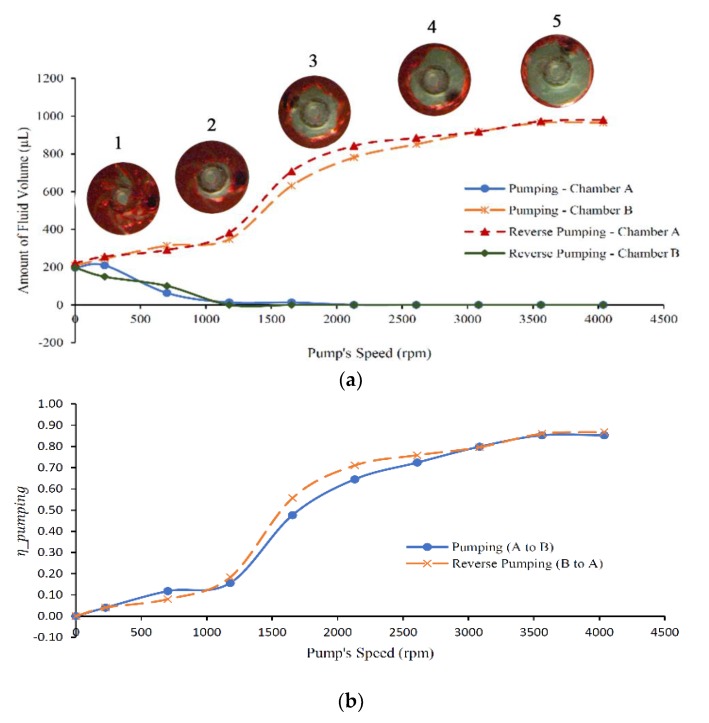
(**a**) Pump speed vs. volume of fluid pumped showing the status of gas volume fraction inside the pumping chamber as the impeller speed increases; and (**b**) pump speed vs. pumping efficiency.

The experimental results of pumping at a range of pumping speed, and pumping efficiency are presented in Figure 8a,b. It is clear from Figure 8a that when the pump speed increases, the volume of liquid increases in the destination chambers (chamber B for pumping and chamber A for reverse pumping), while the liquid level decreases in the suction chambers for both processes (pumping and reverse pumping). The slope change from the 0–1179 rpm and the 1656–4038 rpm regions is due to the sudden change of the gas holdup inside the pump, as described earlier by Schäfer et al. [28]. The rapid change of the fluid from the bubbly regime to the intermittent regime is the main cause of this discontinuity [29]. Pumping chamber images 1 and 2 presented in Figure 8a show an asymmetric distribution of the gas phase (can also be seen in Figure 7c). On the other hand, images 3–5 describe the rotational speeds at which the pumped volume increased significantly and symmetric air distribution can be noticed. The discontinuity could be avoided by initially filling the pump with enough liquid to escape the critical gas volume fraction. Experimental results of pump efficiency shown in Figure 8b prove that the developed centrifugal pump can work in both directions, CW and CCW, in an almost identical pumping performance. The pump can reach a pumping efficiency of 87% when the pump speed is elevated to 4038 rpm. However, the pumping efficiency curve was calculated with the consideration that the accumulated liquid in the destination chamber is always subjected to centrifugal force due to the spinning speed of the microfluidic platform, and there is always a need for a higher pumping force for increasing the volume of pumping fluid. This could be overcome by connecting the pumping chamber and destination chamber by a siphon channel [30]. Some amount of liquid remains in the pumping chamber and cannot be pumped because the majority of the chamber would be filled with air by the time the liquid transfers to pumping chamber B (or to A in the case of reverse pumping), and the pumping efficiency decreases up to a point where it is not enough to pump any liquid. The connecting channels volume is also considered a dead volume since they are always filled with liquid that will not be pumped.

### 3.5. Flow-Switch Pumping 

**Figure 9:**
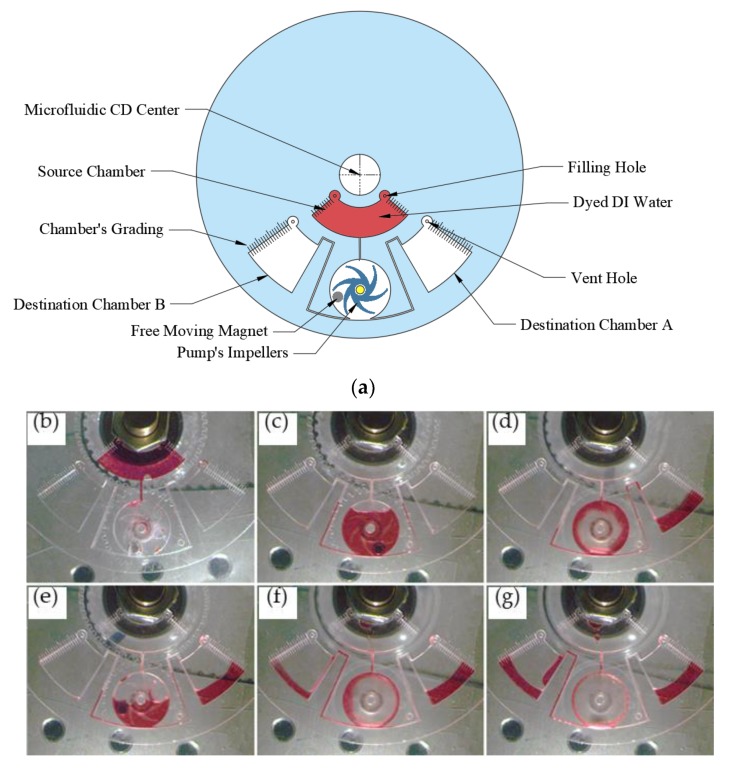
Flow-switch pumping design and experimental results: (**a**) Microfluidic CD design for the flow-switch pumping (**b**–**g**) experimental progress of the flow-switch pumping.

In this test, liquid flow switching is performed on the microfluidic CD using the proposed centrifugal pump. Compared to the previously proposed flow switching mechanisms on microfluidic CD which use active or passive valves [31,32], this work proposes the use of an active centrifugal pump to switch and pump the liquid back towards the microfluidic CD center. Figure 9a illustrates the microfluidic CD design utilized for flow switch along with a pumping application. The design consists of one source chamber, the centrifugal pump, two destination chambers (A and B), and three connecting microchannels to connect the centrifugal pump to the source chamber and the two destination chambers. The suggested design aims to switch and pump the liquid from the source chamber to any of the two destination chambers according to the rotation direction of the centrifugal pump; for instance, when the pump rotates CW, the liquid is pumped towards chamber B, once the direction of rotation is changed to CCW, the liquid flow is switched towards chamber A. The pumped liquid volume can be also accurately controlled by controlling the pumping time, allowing for a volume metering process. 

All the stages of the performed experiment are presented in Figure 9b–g. The process starts with loading the source chamber with 1000 µL of red-dyed water, then spinning the microfluidic CD at 250 rpm to transfer the liquid from the source chamber to the pumping chamber using the centrifugal force (Figure 9b). To pump half of the liquid to destination chamber A, the centrifugal pump is activated CCW at a speed of 1655 rpm (Figure 9c,d). To switch the flow towards destination chamber B, the speed and direction of the centrifugal pump are changed to 3560 rpm and the CW direction. The increase in the pump speed is due to three reasons: (1) the increase of the pumping distance that is longer compared to the previous stage (pumping toward chamber B; (2) the shape of the impeller’s vanes which are facing towards chamber A; and (3) the increase of air volume in the pumping chamber during the pumping toward chamber A. It was noticed that a small amount of liquid is always leftover in the centrifugal pump chamber which cannot be pumped at the implemented pump speed and microfluidic CD speed. To pump this leftover liquid, the microfluidic CD spinning speed was reduced from 200 rpm down to 100 rpm where the liquid was successfully pumped to destination chamber B. 

It is important to mention here that although there are three microchannels connected to the pumping chamber, only one channel can be the pumping port while the pump rotates CW and CCW. This is due to two crucial reasons: (1) the liquid pressure inside the pumping chamber is higher than the outer rim of the microfluidic CD (the position of connection channels to destination chambers) due to the higher centrifugal force. Therefore, the liquid is forced to flow towards the destination chambers and not the source chamber; and (2) the angle of the connection between the connecting channels and pumping chambers is 90 degrees for the source chamber channel, while it is at a tangent for the destination chambers channels. This makes it easier for the liquid to flow towards the destination chambers while the impeller of the centrifugal pump is spinning CW or CCW.

## 4. Conclusions

In this work, a novel centrifugal pump was developed and integrated on microfluidic CDs for active bidirectional liquid pumping. The proposed centrifugal pump partially replicated conventional centrifugal pumps with modification in inlet and outlet positioning to allow the bidirectional pumping option. The pump is developed to run on a dual-motor spinning system where the primary motor controls the spinning of the microfluidic CD while a secondary motor actuates and controls the centrifugal motor. The performance test showed that the developed centrifugal pump can pump a relatively large amount of liquid (around 1 mL) at a volumetric flow rate of 164.93 mm^3^/s and pump rotational speed of 4288 rpm, therefore, it takes 6.06 seconds to pump 1 mL of water. The developed pump was utilized to perform various frequently required microfluidic applications, such as flow reciprocation, bidirectional pumping, and flow switching. The developed centrifugal pump showed the ability to perform real continuous liquid circulation (flow reciprocation) from and to the same chamber while the microfluidic CD is spinning. Results also showed that the developed centrifugal pump can pump 1096 µL of liquid towards the CD center at 87% pumping efficiency while spinning the microfluidic CD at 250 rpm. In conclusion, compared to the previously proposed active pumping mechanism on the microfluidic CD, the proposed centrifugal pump can provide robust, bidirectional, and highly controllable liquid pumping during a spinning or a stationary microfluidic CD. This advanced pumping mechanism can be utilized in any chemical or biological processes on the microfluidic CD without affecting other integrated microfluidic components, such as passive valves or pumps.

## Supplementary Materials

The following are available online at https://www.mdpi.com/2072-666X/11/2/140/s1, Video S1: Continous pumping—Increasing the pump’s speed while the CD is rotating.
